# Primary multidrug-resistant tuberculosis versus drug-sensitive tuberculosis in non-HIV-infected patients: Comparisons of CT findings

**DOI:** 10.1371/journal.pone.0176354

**Published:** 2017-06-06

**Authors:** Duo Li, Wei He, Budong Chen, Pingxin Lv

**Affiliations:** Department of Radiology, Beijing Chest Hospital of Capital Medical University, Tong Zhou District, Beijing, PR China; Universidad Nacional de la Plata, ARGENTINA

## Abstract

**Background:**

Multidrug-resistant tuberculosis has emerged as a global threat. The aim of this work was to compare the CT findings of primary multidrug-resistant tuberculosis and drug-sensitive tuberculosis in non-AIDS adults.

**Material and methods:**

From January 2012 to February 2016, 89 patients with primary multidrug-resistant tuberculosis were retrospectively reviewed, and 89 consecutive drug sensitive TB patients with no history of anti-tuberculous chemotherapy from January 2014 to November 2014 were enrolled as control group. All patients were seronegative for HIV. The patients’ demographic data and the locations, frequency and patterns of lung lesions on chest CT were compared.

**Results:**

Gender and frequency of diabetes were similar between the two groups. The mean age of primary multidrug-resistant tuberculosis patients was younger than that of drug-sensitive tuberculosis (39.0 vs 47.5, P = 0.005). Lung cavitary nodules or masses were more frequently observed and also showed greater extent in primary multidrug-resistant tuberculosis compared with drug-sensitive tuberculosis. The extent of bronchiectasis was significantly greater in primary multidrug-resistant tuberculosis than in drug-sensitive tuberculosis. Calcification, large nodules and calcified lymph nodes were more frequent in drug-sensitive tuberculosis.

**Conclusion:**

Characteristic chest CT findings may help differentiate between primary multi-drug resistant tuberculosis and drug-sensitive tuberculosis in patients without HIV infection.

## Introduction

Multidrug-resistant tuberculosis (MDR-TB) refers to tuberculous infection caused by acid-fast bacterial (AFB) organisms resistant to at least two drugs, isoniazid and rifampin[1]. In 2014, there were an estimated 480,000 new cases of MDR-TB worldwide, and approximately 190,000 deaths from MDR-TB[2]. Early identification of patients with DR-TB increases the likelihood of treatment success and interrupts transmission.

MDR-TB is divided into primary MDR-TB and acquired MDR-TB. Acquired MDR-TB refers to resistance developed during or following chemotherapy in patients who had previously been regarded as DS TB[3]. Primary MDR-TB characterizes patients who have no prior TB treatment history or treatment of <1 month[4]. A national survey of drug-resistant tuberculosis in China revealed a serious epidemic of drug-resistant tuberculosis. In 2007, an estimated 110,000 incident cases of MDR tuberculosis including 86,000 cases of primary MDR and 24,000 cases of acquired MDR tuberculosis were diagnosed in China[5]. Thus, most cases of MDR tuberculosis in China result from primary transmission.

Several studies have shown that multiple cavities and findings of chronicity such as bronchiectasis and calcified granulomas are more common in patients with MDR TB[4, 6]. However, these findings may reflect the chronic course of acquired MDR TB. The imaging features associated with chronicity or the TB healing process may not be common in patients with primary MDR TB[7]. In primary MDR-TB, the two most frequent abnormal CT patterns are tree-in-bud sign and acinar nodule[8]. The potential of radiologic evidence to indicate MDR—TB merits investigation because of the rapid availability of CT. Unfortunately, few studies have compared imaging findings of primary MDR TB and DS TB. One study with a small sample size that specifically compared image differences between these two groups noted that bilateral involvement of parenchymal lesions and multiple cavities were significant CT findings associated with primary MDR TB rather than drug-sensitive TB (DS-TB)[7]. Here, we perform a further study using an expanded sample size.

## Material and methods

### Patients

From January 2012 to February 2016, 95 patients who were diagnosed as primary MDR TB (disease that developed in patients with no history of anti-tuberculosis chemotherapy or therapy history of less than one month) and had a CT scan available were enrolled. Among the 95 patients, three patients had coexisting lung cancer, one patient had coexisting silicosis, one patient had lobectomy and one patient had complete atelectasis due to pneumothorax. These patients were excluded, and 89 patents were finally enrolled in the study.

For comparison, 89 consecutive primary DS TB (defined as disease with no resistance to any drug and no history of anti-tuberculous chemotherapy) patients from January 2014 to November 2014 were also enrolled. All CT scans were performed within 30 days of TB diagnosis. All patients enrolled in this study were inpatient and HIV-seronegative. The Institutional Review Board of Beijing Chest Hospital Affiliated to Capital Medical University approved this study. The need for informed consent was waived because this was an observational retrospective study and all patient data were analyzed anonymously.

### Chest CT

Chest CT was performed using one of two multi- detector CT scanners: Light Speed VCT (General Electric Medical Systems, Milwaukee, WI, USA) or Optima CT 680 (General Electric Medical Systems, Milwaukee, WI, USA). The scanning parameters were as follows: 120 kV tube voltage; automatic tube current modulation; detector collimation, 64 × 0.625 mm; rotation time, 500 ms; pitch, 1.375. The raw data were reconstructed into axial images with a 5-mm slice thickness at 5-mm intervals. For thin-section CT, axial images with a 1.25-mm slice thickness at 1.25-mm intervals and a high-spatial-frequency algorithm were obtained. The scan data were directly displayed on monitors of the Picture Archiving and Communication System (PACS). On the monitors, both mediastinal (window width, 400 H; window level, 20 H) and lung (window width, 1,500 H; window level, -700 H) window images were available for analysis.

### CT imaging evaluation

Two radiologists reviewed all CT images in a blinded manner, and decisions were reached by consensus. The presence of each parenchymal abnormality pattern in each lobe (six parts: right upper lobe, right middle lobe, right lower lobe, upper division of left upper lobe, lingular division of left upper lobe, and left lower lobe) was recorded. The assessed patterns of parenchymal abnormalities included: tree-in-bud and small centrilobular nodules (small centrilobular nodules <10 mm in diameter and branching nodular structures), large nodules (10–30 mm in diameter), ground glass opacity (GGO), consolidation (lobular or sub-segmental, segmental or lobar), cavities (cavities in consolidation, cavitary nodule or mass), bronchiectasis, calcification (including nodules with calcification) and atelectasis. In addition, the presence of mediastinal or hilar lymph node enlargement (with and without calcification), pleural effusion and pericardial effusion was recorded. Each lung lobe was evaluated with regard to the presence or absence of each parenchymal abnormality. The laterality (unilateral or bilateral) of lung lesions was also recorded. The extent of CT findings was calculated as the number of lobes involved.

### Microbiology evaluation

The smear slides were stained using the Ziehl-Neelsen method according to the World Health Organization (WHO) protocol [9]. The sodium hydroxide method for the culture of *M*. *tuberculosis* isolates was performed according to previously described procedures[10].The standard Löwenstein-Jensen(LJ) proportion method for the drug-sensitive test (DST) of *M*. *tuberculosis* isolates was performed according to previously described procedures[11].The critical concentrations of the tested drugs were 0.2μg/ml for isoniazid and 40μg/ml for rifampicin. During DST, quality control was routinely performed using the reference strain H37Rv.

### Statistical analysis

Statistical analyses were performed using commercially available software (SPSS17.0). Male-to-female ratios and the presence of each pattern of parenchymal abnormality in primary MDR TB and DS TB were evaluated using the chi-square test or Fisher's exact test. Differences in age and the number of lobes involved in parenchymal abnormality were compared using the Mann-Whitney U test. Stepwise logistic regression analysis was performed to determine how independently useful CT features were in distinguishing between primary MDR and DS TB. A P value lower than 0.05 was considered significant.

## Results

The demographics of primary MDR TB and DS TB patients are summarized in Table 1. The mean age of the MDR TB patients (39.0 years, range from 12 to 81 years) was younger than that of DS TB patients (47.5 years, range from 16 to 86 years) (P = 0.005). The gender ratio was not significantly different between patients with primary MDR and DS TB (P = 0.63). Males were predominant in both groups: 65.2% of primary MDR TB patients and 68.5% of DS TB patients were male. The prevalence of diabetes did not differ significantly between the two groups.

**Table 1:** Demographics of patients with primary MDR TB and DS TB^a^.

		Primary MDR TB	DS TB	P*
Age (years) ^b^		39.0±17.3	47.5±20.1	<0.01
Gender, n (%)				0.63
	Male	58(65.2)	61(68.5)	
	Female	31(34.8)	28(31.5)	
Diabetes Mellitus, n (%)		27(30.3)	29(32.6)	0.75

^a^ Values are given as the number of patients(%), unless otherwise indicated

^b^ Values are given as the mean±SD

*Age was compared using the Mann-Whitney U test. Gender and diabetes mellitus were compared using the Pearson *X*^2^ test.

**Fig 1:**
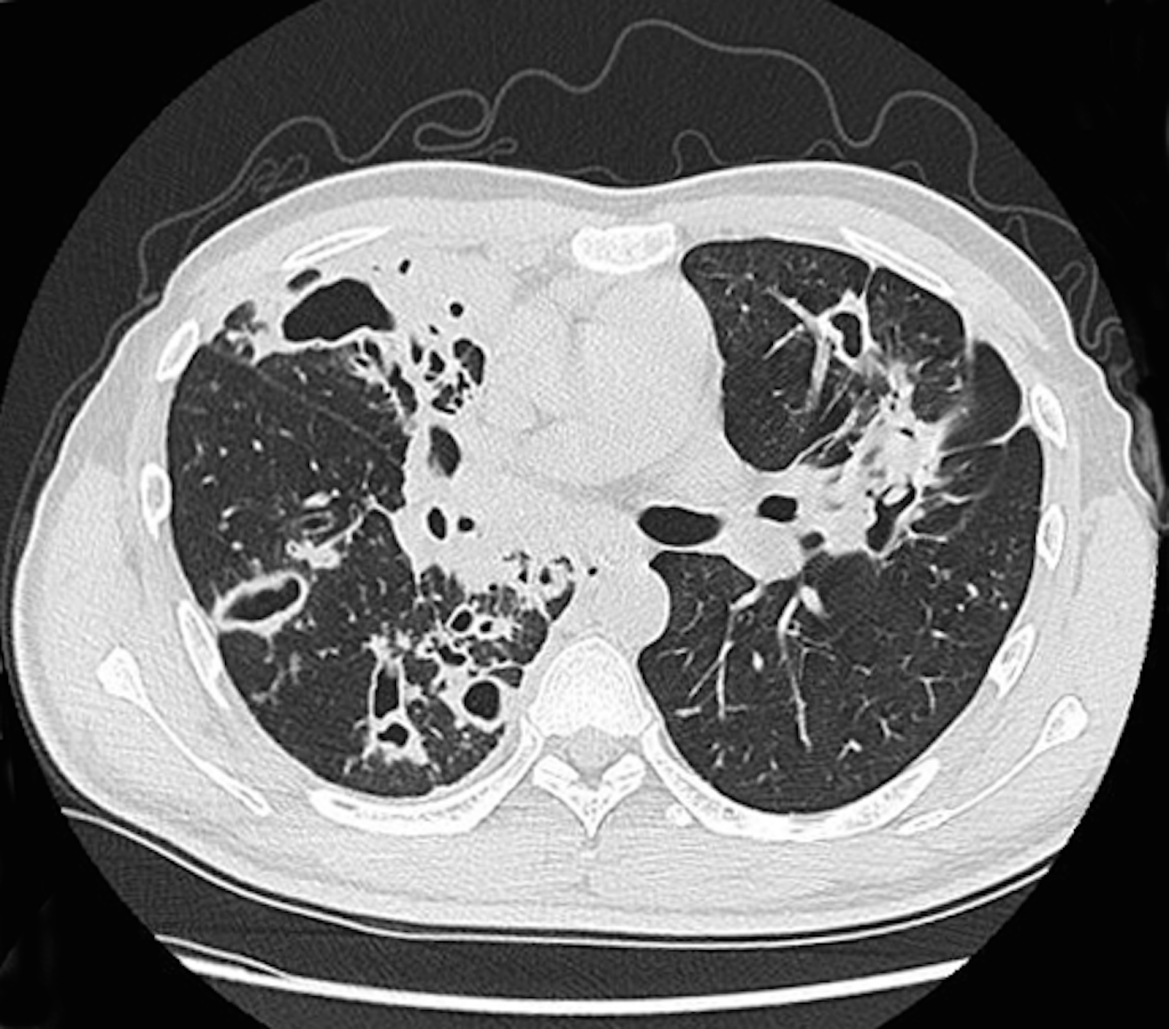
Primary multidrug-resistant TB in a 29-year-old man. A lung window of a transverse thin-section CT scan (1.25-mm-section thickness) showing multiple cavities in both lungs.

**Fig 2:**
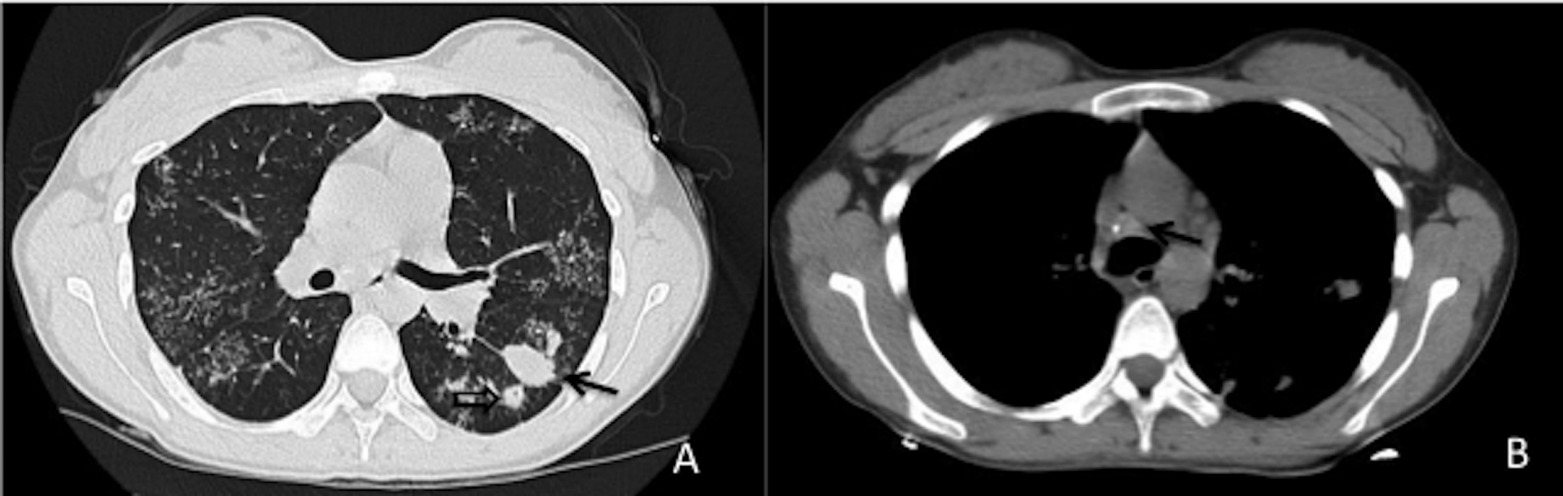
Drug-sensitive TB in a 20-year-old woman. A. Lung window of a transverse thin-section CT scan (1.25-mm-section thickness) showing a large nodule (arrow) and small nodules (empty arrow) in the left lower lobe. Tree-in-buds were also observed on both sides. B. Mediastinum windows of a transverse CT scan (5.0-mm section thickness) showing an enlarged lymph node with calcification (arrow).

The CT findings of primary MDR and DS TB are summarized in Table 2. Cavitary nodules or masses were more frequently observed in primary MDR TB than in DS TB (57.3% VS 38.2, P = 0.01) (Fig 1). By contrast, large nodules, calcification and calcified lymph nodes were more frequently observed in DS TB than in MDR TB (large nodules, 22.5% VS 11.2, P = 0.045; calcification, 43.8% VS 28.1%, P = 0.03; calcified lymph nodes, 19.1% VS 2.2%, P < 0.001) (Fig 2). Other CT findings did not differ significantly between primary MDR and DS TB.

**Table 2:** Comparison of the CT Findings for primary MDR and DS TB ^a^.

CT findings	Primary MDR TB (n = 89)	DS TB (n = 89)	P*
Tree-in-bud pattern and Centrilobular nodules	85(95.5)	87(97.8)	0.41
Large nodule	10(11.2)	20(22.5)	0.045
Consolidation			
Lobular or sub-segmental	79(88.8)	85(95.5)	0.10
Segmental or lobar	33(37.1)	31(34.8)	0.76
Cavity			
Cavity in consolidation	43(48.3)	42(47.2)	0.88
Cavity nodule or mass	51(57.3)	34(38.2)	0.01
GGO	22(24.7)	30(33.7)	0.19
Bronchiectasis	62(69.7)	50(56.2)	0.06
Calcification	25(28.1)	39(43.8)	0.03
Atelectasis	16(18.0)	9(10.1)	0.13
Lymphadenopathy			
Mediastinum	24(27.0)	30(33.7)	0.33
Hilar	14(15.7)	18(20.2)	0.44
Density of the LN			
Calcified lymph node	2(2.2)	17(19.1)	<0.001
Non calcified lymph node	23(25.8)	14(21.3)	0.48
Pleural effusion	26(29.2)	21(23.6)	0.40
Pericardial effusion	3(3.4)	7(7.9)	0.19
Laterality			0.35
Unilateral	20(22.5)	15(16.9)	
Bilateral	69(77.5)	74(83.1)	

^a^ Values are given as the number of patients(%).

*Values are derived from the Pearson chi-square test.

There are complete data in each group (n = 89) for all variables.

**Fig 3:**
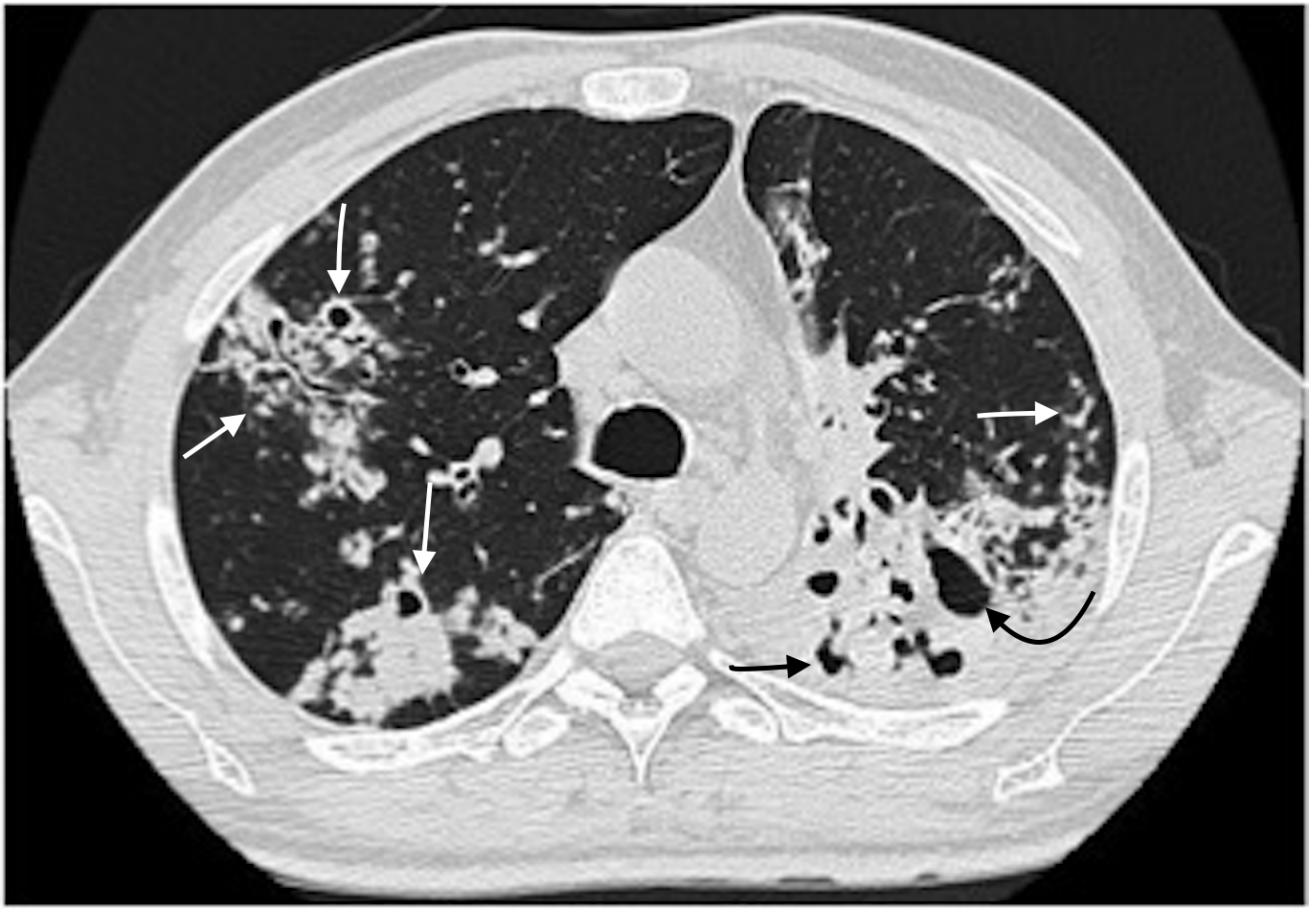
Primary multidrug-resistant TB in a 39-year-old man. Lung window of a transverse thin-section CT scan (1.25-mm-section thickness) showing multiple consolidations, small centrilobular nodules, tree-in-buds and bronchiectasis (arrow). A cavity in consolidation at the left lower lobe was also noted (curved arrow).

Unilateral or bilateral involvement of parenchymal lesions and the number of lobes involved were not statistically significant difference between primary MDR and DS TB. The extent of pulmonary abnormalities in MDR TB and DS TB is summarized in Table 3. The extent of cavity and bronchiectasis were significantly greater in MDR TB than in DS TB (Fig 3). The extent of large nodules and calcification were significantly smaller in MDR TB than in DS TB. Other CT features did not differ significantly between MDR TB and DS TB.

**Table 3:** Comparison of the extent of pulmonary abnormalities in pMDR and DS TB ^a^.

Characteristics	Primary MDR TB (n = 89)	DS TB (n = 89)	P*
Total	4.44	4.43	0.78
Tree-in-bud pattern and Centrilobular Nodules	3.57	3.7	0.68
Large nodule	0.13	0.31	0.04
Consolidation			
Lobular or sub-segmental	2.22	2.48	0.17
Segmental or lobar	0.55	0.47	0.65
Cavity			
Cavity in consolidation	0.96	0.79	0.52
Cavity nodule or mass	1.03	0.64	0.01
GGO	0.46	0.54	0.24
Bronchiectasis	1.70	1.13	0.02
Calcification	0.55	0.73	0.047
Atelectasis	0.29	0.15	0.12

^a^ Values are given as the number of pulmonary lobes involved by each specific abnormality.

*Values are derived from the Mann-Whitney U test.

There are complete data for all variables in each group (n = 89).

Stepwise logistic regression was performed used the forward LR selection method. The dependent variable was the type of TB (DS-TB or MDR TB). The covariant variables including the variables in Tables 1–3 that differed significantly between the two groups (age, large nodule, cavity nodule or mass, calcification, calcified lymph node, extent of large nodule, extent of cavity nodule or mass and extent of bronchiectasis). The result was illustrated in Table 4. The age (odds ratio, 0.98; 95% confidence interval 0.96–1.00; P = 0.03), extent of cavities (odds ratio, 1.26; 95% confidence interval 1.02–1.56; P = 0.03) and absence of calcified lymph nodes (odds ratio,8.96; 95% confidence interval1.94–41.28; P <0.01) were associated with primary MDR TB.

**Table 4:** Logistic regression analyses to identify the variables that associated with primary MDR TB.

Variable	OR	95%CI	P
Absence of calcified lymph nodes	8.95	1.94–41.29	<0.01
Extent of cavities	1.26	1.02–1.56	0.03
Age	0.98	0.96–1.00	0.03

OR, odds ratio; CI, confidence interval

## Discussion

The only previous study comparing primary MDR TB and DS TB had 39 patients in each group [7]. Our study presents the CT findings of primary MDR TB vs. DS TB in non-HIV infected patients in a tuberculosis endemic country. This is the largest such comparison to date. Our study demonstrated that cavitary nodules or masses were more frequently observed in primary MDR TB than in DS TB and that the extent of cavities and bronchiectasis was greater in primary MDR TB than DS TB. Large nodules, calcification and calcified lymph nodes were more frequently observed in DS TB than in MDR TB.

In our study, patients with primary MDR TB were significantly younger than those with DS TB, whereas gender did not differ between the two groups, consistent with the results of previous studies[4, 12]. The proportion of patients with diabetes did not differ significantly between the two groups, consistent with reports suggesting that diabetes mellitus is not associated with drug-resistant TB[13, 14].

The presence of cavities has been noted as more common for MDR than DS TB[4, 6, 12, 15]. However, most of these studies have included both acquired MDR and primary MDR TB. Limited drug penetration into cavities, which harbor large numbers of mycobacteria, is believed to contribute to the development of acquired drug resistance TB[16]. However, in our study and Yeom et al’s study[7], cavities were more frequently observed in patients with primary MDR TB than in those with DS TB. The mean number of cavities in patients with primary MDR TB was greater than that in patients with DS TB in Yeom et al’s study[7]. Instead of counting the number of cavities, our study calculated the lobes involved with cavities, and the extent of cavities was greater in primary MDR TB than in DS TB in our study. The mechanism underlying the greater number of cavities in primary MDR TB than in DS TB remains unclear. Microorganism virulence and patient immune status may be involved in the development of multiple cavities[7].

Reports including both acquired MDR TB and primary MDR TB have observed that bronchiectasis is more frequent in MDR TB than in DS TB[12]. However, a previous study comparing primary MDR with DS TB[7] found no significant difference in bronchiectasis between the two groups, similar to our results. However, our study demonstrated that the extent of bronchiectasis was significantly greater in MDR TB than in DS TB.

Centrilobular nodules or tree-in-bud appearance and lobular or sub-segmental consolidations were the most frequent CT abnormalities in both the MDR and DS TB groups. The frequency and extent of these CT findings did not differ significantly between these two groups in our study, consistent with previous reports[4, 6, 8, 12, 14, 17]. Centrilobular micronodules and tree-in-bud appearance are the most common CT findings of active pulmonary tuberculosis[18]. Pathologically, these CT features correspond to the bronchogenic spread of caseation necrosis materials and granulomatous inflammation caused by tuberculosis[19, 20].

In previous reports, bilateral involvement was more commonly observed in patients with MDR-TB than those with DS TB[7, 15]. The failure to observe a difference in the extent of involvement between the two groups in our study may be due to the enrollment of inpatients, who have more serious symptoms and radiological findings than outpatients.

Previous reports indicated that calcified granulomas are more common in patients with MDR TB because the majority of cases were due to acquired MDR[4, 6]. However, a report comparing the CT findings of primary MDR and DS TB found that calcification is more common in DS TB (18%) than in primary MDR TB (13%), although this difference was not statistically significant[7]. This study is the first to report that calcification, large nodules and calcified lymph nodes were more frequently seen in DS TB than in primary MDR TB. Further prospective study is needed to testify the trues.

This study has limitations. The study might have involved some selection bias because not all primary MDR TB patients underwent CT scans in our hospital and not all patients with DS TB in the same period were enrolled. Second, this study was conducted at a single specialized TB hospital in Beijing, and thus the results might not reflect the overall situation in the region. This hospital is the largest specialized hospital for TB, and we enrolled only inpatients; consequently, the inclusion rate of serious TB cases may have been high. This selection bias might have led to the inclusion of more serious lesions than in previous reports.

In conclusion, more extensive cavities and bronchiectasis were observed in patients with primary MDR TB, whereas calcification, large nodules and calcified lymph nodes were more frequently observed in DS TB. These CT imaging findings may help differentiate primary MDR TB from DS TB.

## Supporting information

S1 Dataset The row data of all patients. (XLS)
